# Rapid authentication of pharmaceuticals via DNA tagging and field detection

**DOI:** 10.1371/journal.pone.0218314

**Published:** 2019-06-13

**Authors:** Lawrence Jung, Michael E. Hogan, Yuhua Sun, Benjamin Minghwa Liang, James A. Hayward

**Affiliations:** Applied DNA Sciences, Inc. Stony Brook, New York, United States of America; University of Houston, UNITED STATES

## Abstract

A small PCR-generated DNA fragment was introduced into a pharmaceutical grade ink as a molecular taggant, and the DNA tagged ink was delivered onto the surface of capsules by standard high-speed offset printing. The amount of DNA in the ink on each capsule is roughly 10^−12^ fold lower than that allowed as safe by the United States Food and Drug Administration (FDA) and the WHO with regards to acceptable limits of residual DNA. The printed ink on the capsule surface was sampled by swabbing, followed by direct analysis of the DNA-swab complex, without subsequent DNA purification. It was shown that DNA recovered from the ink by swabbing was suitable for PCR-CE analysis—a widely used method in forensic science and was also suitable for qPCR and isothermal DNA amplification, when coupled with portable devices similar to those used for environmental sampling and food safety testing. The data set a precedent: A small DNA fragment could be introduced as an excipient into a pharmaceutical application, and thereafter tracked through the pharmaceutical supply chain via forensic DNA authentication.

## Introduction

Counterfeit pharmacological agents are a threat to the drug supply and are estimated to be found in the marketplace at greater than 20%, especially in developing markets [1, 2, 3]. Present methods to verify real versus counterfeit drug in the field, rely on packaging verification and serialized barcoding [3, 4]. More recently, field deployable analytical methods have been described, based on the use of hand-held Raman spectroscopy and the like. However, the Raman approach reveals only the principle components (generally the excipients) of the dosage formulation and in most cases, cannot detect the active agent, nor can it identify the quality of the components of the drug formulation [3, 4, 5].

**Fig 1:**
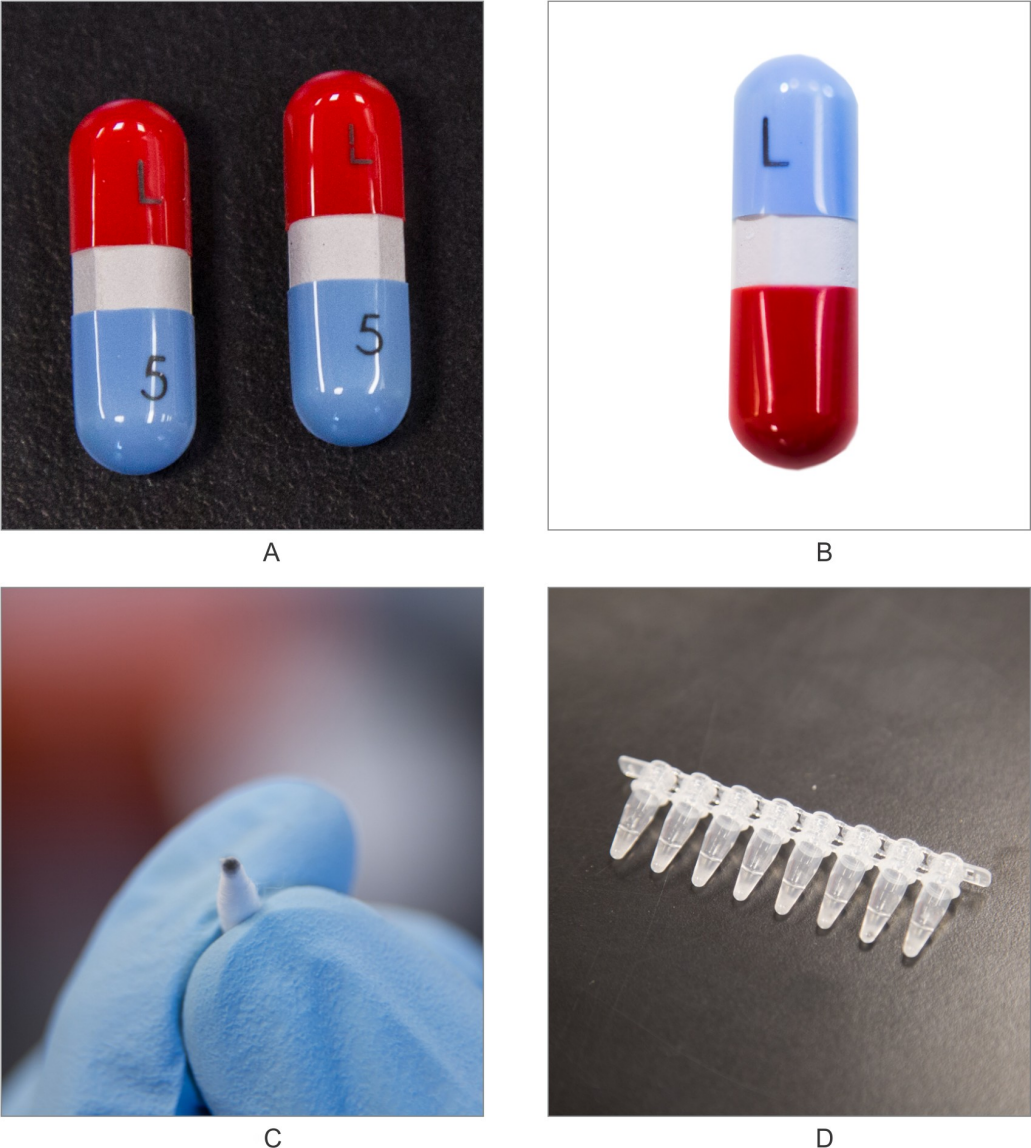
Methods for tagging ink (OPACODE S-1-17823 black) with DNA then applying to acetaminophen capsules in August 2014. (A) two ink-labeled acetaminophen capsules are displayed. Tagging was performed in 2014. The capsule on the left was DNA tagged in this pilot (with both an “L and a “5”) using standard high-speed capsule pad printing. The matched capsule on the right was tagged with the same “L” and “5” but without DNA in the black ink. Both the “+DNA” and “–DNA” capsules appear identical to the eye. (B) It can be seen that the DNA-tagged ink can be swabbed from the surface using ethanol as a wetting solvent. Also note, there is no marring of the surface of the capsule in the process. (C) The swab tip after swabbing of the capsule, with the [DNA+ink] complex positioned at the tip. (D) 0.2mL tubes for PCR amplification each contain the tip of a cotton swab (with DNA containing ink on it) to be analyzed via PCR/CE analysis (Fig 2), or via qPCR amplification and detection (Fig 3), or isothermal amplification and detection (Fig 4).

Here, as an alternative to serialized barcoding and hand-held spectroscopy, we describe the use of small, biochemically generated DNA fragments as a “functional” excipient or “molecular barcode” to be introduced into and recovered from a drug formulation: the goal is to build the bar code into the drug itself (Fig 1).

It is well known that DNA constitutes the molecule that is used to store the information required to support life. In humans, that information differs enough among individuals in that the DNA in each individual may be used as a “molecular tag” for the purposes of identifying criminals or familial relationships: based upon variation in the natural DNA tag within us. Such technology for “DNA-ID” has become sophisticated and is presently a core technology in forensic science. [6, 7, 8, 9, 10]

Recently, we have developed a technology to produce small DNA fragments by PCR at an industrial scale [11]. The DNA is manufactured with purified enzymes to yield DNA fragments < 200bp, which both the FDA and the World Health Organization (WHO) have taught are too small to be considered to be a gene but still long enough to encode enough information to be used as a DNA-based “molecular bar code”, similarly, there are billions of different alpha-numeric combinations possible for any ordinary ink bar code, similarly, there are billions of different combinations of nucleic acid sequences possible to generate “DNA” barcodes from small DNA fragment produced enzymatically [12, 13, 14, 15, 16, 17, 18].

**Fig 2:**
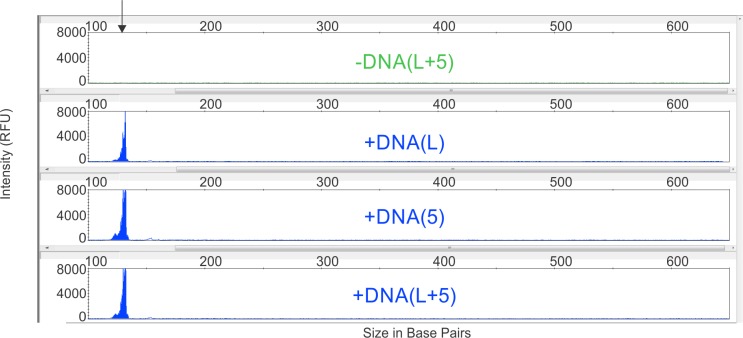
PCR and CE analysis–Confirmatory testing for field-deployable nucleic acid analysis. This is a display of the final output of the analysis by PCR/CE, with clear differentiation between DNA tagged (+DNA) and untagged (-DNA) capsules. The DNA of interest is less than 200 base pairs long and appears as a single discrete peak. The PCR-CE data also confirms that there is only one discreet DNA of interest in the capsule. In the (-DNA) capsule, where the “L “and “5” have both been swabbed off for analysis denoted as “-DNA (L+5)” there is no DNA peak observed at the appropriate area of the CE trace. On the other hand, for the DNA tagged capsules (+DNA) where the “L,” “5” or both “L + 5” have been sampled, DNA is detected at the correct size as indicated by the blue peaks without spurious amplification nor additional peaks. These specimens were collected by swabbing and analyzed in 2016, more than 2 years after the DNA-tagged capsules were manufactured.

As a first step towards the development of a DNA-tagging approach that is suitable as an “internalized DNA bar code”, we describe here a pharmaceutical pilot study focused on the introduction of a DNA fragment smaller than 200 base pairs long (Fig 2B) directly into pharmaceutical grade ink, which is then used for high speed printing of alphanumeric symbols onto the surface of capsules and tablets.

## Materials and methods

### Samples

The DNA tagging of acetaminophen capsules was performed in August 2014, at a local over-the-counter (OTC) generics pharmaceutical manufacturer in Long Island, New York. To perform the tagging, a 5 mL DNA concentrate—was inoculated into one liter of pharmaceutical grade OPACODE S-1-17823 black ink and run through a R. W. Hartnett, Model B-15-8 printer for direct printing onto acetaminophen capsules. Samples of non-tagged acetaminophen capsules were purchased OTC at local pharmacies. As seen in Fig 1B, there is virtually no difference in appearance between the two capsules. All DNA tagged, printed capsules were placed on long-term stability.

### Sampling methods

Capsules were swabbed with a Puritan 6 Inch Sterile Tapered Mini-tip Cotton Swab with Wooden Handle. This type of swab is commonly used by medical professionals, engineers, and artists and can be wetted by a solvent such as ethanol, isopropanol, methyl ethyl ketone with equivalent results. The results shown in Fig 2 deployed only ethanol. These solvents dissolve only the tagged ink, they do not dissolve nor damage the capsule. The tips of these swab samples were then clipped for direct insertion into the 0.2 mL PCR reaction tube. All samples were tested between the marking of the capsules in August 2014 to March 2017.

### Pre-Screening Utilizing PCR and CE Methods

PCR thermocyclers utilized for the tests were either the Applied Biosystems 2720 Thermal Cycler or SimpliAmp Thermal Cycler by Thermo Fisher. The PCR Master Mix recipie can be found in Table 1. PCR primers were purchased from Integrated DNA Technologies, where the forward primer was labeled with either FAM or HEX. For the electropherograms in Fig 2B, HEX labeled primers were used, though to keep the figure colors consistent, the DNA containing traces have been changed to blue from green. The thermocycling parameters were: 1st step at 95.0°C for 3 minutes followed by 32 cycles of: 94.0°C denature for 20 seconds (sec), anneal at 48.0°C for 20 sec, and elongation at 72.0°C for 20 sec. This was followed by a final elongation step at 72.0°C for 5 minutes (min) and a 4.0°C hold.

**Table 1:** The composition of the reagent Mix for the Polymerase Chain Reaction Assay.

PCR assay reaction mix component	Per Tube (μL)
Extract-N-Amp PCR ReadyMix	10.0
Extraction Solution	2.0
Dilution Solution	2.0
10 μM Forward Primer	0.5
10 μM Reverse Primer	0.5
PCR Certified Water H_2_0	3
25 mM MgCl_2_	2
Total Volume	20

For capillary electrophoresis (CE), the Applied Biosystems Instruments 3130xL and the 3500xL were both used interchangeably with equivalent results. Polymer POP-7 was used with a 36 cm array, using 1x Genetic Analyzer Buffer with EDTA and deionized water. The analysis solution per well includes 10 μL of HiDi, 0.125 μL of Liz 600 size standard, with 1 μL of PCR product. The instrument used for the electropherograms in Fig 2 was the 3130xL; the instrumentation and results are representatively summarized in Fig 2.

### Real time quantitative PCR (qPCR) and detection

qPCR reagents, TaqMan Fast Advanced Master Mix; [19] also a GE Life Sciences, illustra PuReTaq Ready-To-Go PCR Beads was evaluated [20]. qPCR was performed using the reagents and protocols from the vendor. The TaqMan Probe and primer mix was manufactured by Thermofisher using their Custom TaqMan Gene Expression Assay [21].

The qPCR device utilized in this study was the MyGo Mini, due to the fact it has an open tangential optical path which makes it suitable for direct analysis of swabs and other solid materials introduced directly into the qPCR tube’s optical path [22].

**Fig 3:**
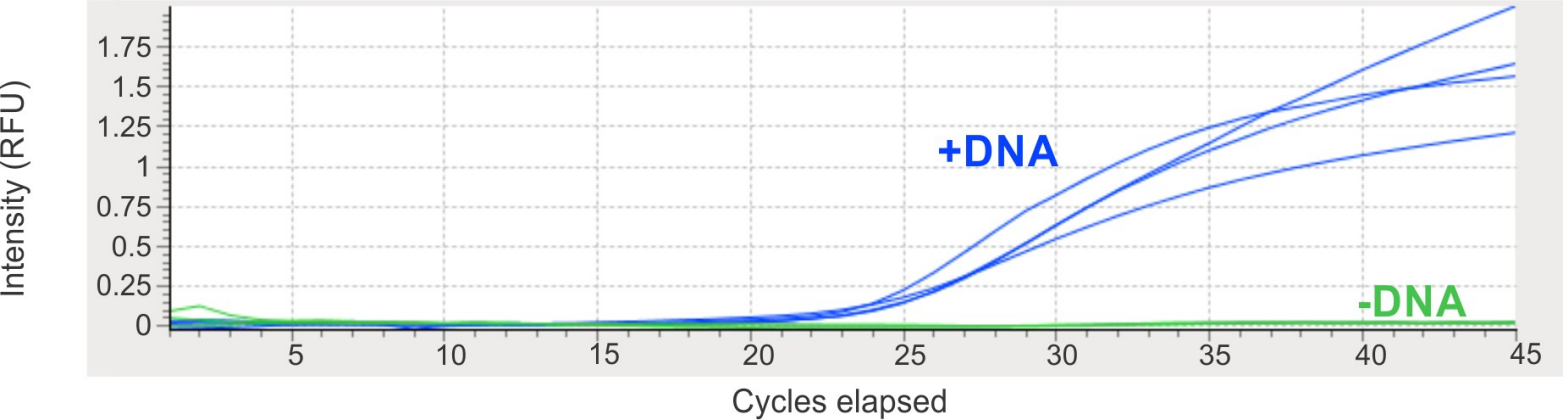
Field-deployable nucleic acid analysis—Real time, qPCR. This is a graphical representation a molecular probe qPCR assay. This can be accessed and viewed on a laptop or PC. As depicted here, there is a clear differentiation in the amplification curves between the DNA tagged (+DNA) capsules versus the non-DNA tagged (-DNA) capsules with minor sample to sample variation. In all cases both the “L” and “5” symbols have been swabbed. As seen here, clear differentiation between tagged and untagged could be obtained by 30 cycles (25 minutes) but since we wanted to show a plateau, the reaction has been taken to completion (45 cycles). The software generates a table from such data where a human interpreter is required to call the sample as containing DNA. Work is in progress to introduce a user interpretation-free algorithm producing a simple yes/no output. These specimens were collected by swabbing and analyzed by qPCR in March 2017, more than 2 years after the DNA-tagged capsules were manufactured.

qPCR was performed according to kit specifications. For each sample, using the GE Pellet process, the master mix was used for resuspension of two freeze-dried reagent pellets in frosted 0.1 mL flip cap tubes with a 50 μL of reagents total volume per tube, the composition of which is shown in Table 2. This is a precursor step to a lyopholizable kit that can make an in-field system more practical. The sample used for analysis consists of a cutting of the tip of a cotton swab after swabbing of the tagged pharmaceutical. The reaction then underwent thermocycling utilizing a MyGo Mini for 40 cycles of a 2 cycle PCR at 95°C for 10 sec and 60°C for 30 sec, with the acquisition taking place at the 60°C step [22]. This assay takes approximately one hour for the amplification and detection process. The instrumentation and results are summarized in Fig 3.

**Table 2:** The composition of the Master Mix for the GE illustra PuReTaq Ready-To-Go PCR Beads (Panel A) and Thermo Fisher’s FastTaq Ready Mix (Panel B).

Panel A: GE illustra PuReTaq Ready-To-Go PCR Beads		Panel B: Thermo Fisher’s FastTaq Ready Mix	
PCR reaction mix component	Per Tube	PCR reaction mix component	Per Tube
Custom TaqMan Gene Expression Assay	1.5 μL	Custom TaqMan Gene Expression Assay	6.25 μL
PCR Grade H20	48.5 μL	PCR Grade H20	18.75 μL
GE illustra PuReTaq Ready-To-Go PCR Beads	2 pellets	FastTaq	25 μL
Total Volume	50 μL	Total Volume	50 μL

The results in Fig 3 were based on MyGo mini instrument with GE master mix reagents on ethanol-wetted swabs. Additional testing that utilized combinations of technologies listed in Table 2 and materials and methods showed confirmatory results and thus were not included in this study.

### Recombinase Polymerase Amplification (RPA) and detection

**Fig 4:**
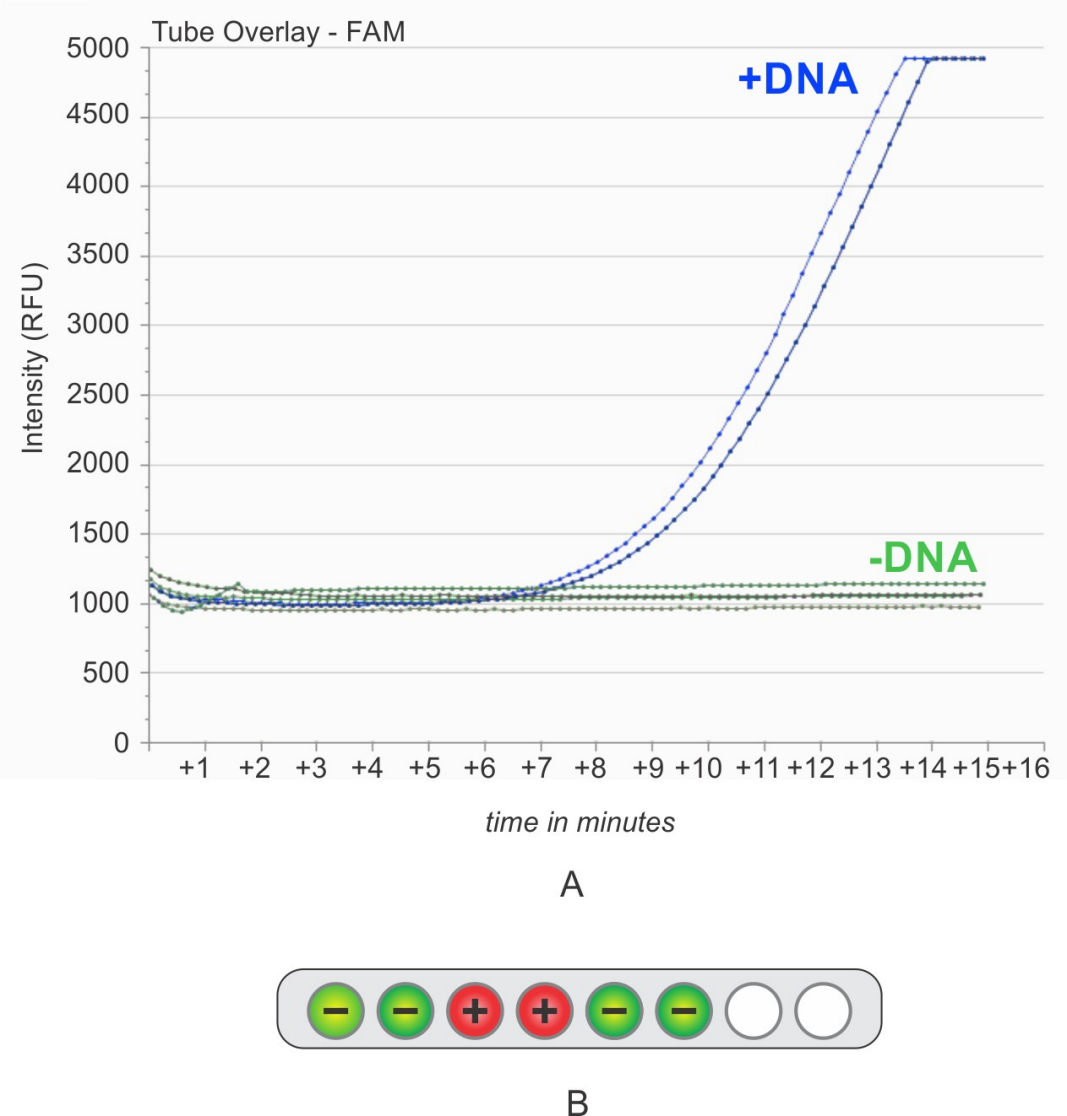
Field-deployable nucleic acid analysis—Isothermal DNA amplification and detection. Isothermal amplification block and detector was deployed using RPA real time quantitative isothermal amplification detection chemistry. (A) A representative real-time amplification data derived from the assay as deployed for direct analysis of a swab-DNA complex. As shown, there is a clear differentiation in the amplification curves of the DNA tagged (+DNA) capsules (“L+5” sampling) versus the untagged (-DNA) capsules (“L+5” sampling), with only minor sample to sample variation. As seen here, the differentiation between tagged and untagged could be called and stopped within 10 minutes, but for this assay (since we wanted to show a plateau), it has taken 15 minutes to achieve for completion. (B) Positive and negative results shown graphically as a Positive (+) or a Negative (–) on the machine display. This allows the removal of the user interpretation, and allows the machine to have learned a pattern and call a result as is. These specimens were collected by swabbing and analyzed by isothermal methods in 2016, more than 2 years after the DNA-tagged capsules were manufactured.

RPA assays were conducted after measuring the DNA content of the capsules via better known PCR/CE methods. The RPA kit was purchased from TwistDx. The RPA isothermal amplification procedure was performed using the reagents and protocols from the TwistAmp exo kit [23]. A custom TwistAmp exo assay was developed for the DNA tag used in this study and was provided by TwistDx as a freeze-dried kit (Table 3). This kit is then developed into a two-part system: a vacuum sealed pouch with 0.2 mL tubes containing custom freeze-dried pellets and a tube of custom buffer where neither part requires refrigeration. An ethanol wetted swab tip is cut, then placed into the 0.2 mL isothermal reaction tube containing the custom freeze dried pellet and then rehydrated with 50 μL of the custom buffer, as per the standard protocols in the standard TwistAmp exo kit [23]. The reaction then incubated in an Axxin T8-ISO at 38°C for 15 min. Fluorescence measurements were taken every 26 seconds [24]. The instrumentation results are representatively summarized in Fig 4.

**Table 3:** The composition of the master mix for the Customized TwistAmp exo assay as equivalent to the manual^23^.

TwistAmp exo assay reaction mix component	Off the Shelf	Custom Assay
	Per Tube (μL)	Per Tube (μL)
Standard Freeze Dried Pellet Components	N/A	N/A
10 μM AF Primer	4.2	N/A
10 μM AR Primer	4.2	N/A
10 μM D Primer	4.2	N/A
10 μM Exo probe	1.2	N/A
Rehydration Buffer	29.5	29.5
PCR Grade H20	0.7	14.5
280mM MgAc	6	6
**Total Volume**	**50**	**50**

This isothermal amplification approach can significantly reduce assay time. If paired with a device that includes an algorithm that can provide a positive or negative result, it can reduce user interpretation. The fact that portable, battery powered, network enabled devices, are being developed by multiple manufactures makes this solution attractive to lay users.

## Results and discussion

In August 2014, we conducted a pilot DNA tagging study of acetaminophen capsules. In this study, the pharmaceutical grade ink used to tag the capsule exterior was formulated with a DNA tag. To execute the study, a 5 mL concentrate of that DNA was inoculated into one liter of a well-known food grade pharmaceutical ink (OPACODE S-1-17823 black ink) and run through a R. W. Hartnett, Model B-15-8 high speed rotogravure printer for direct printing onto acetaminophen capsules. Matched samples of un-tagged acetaminophen capsules were purchased from local pharmacies. As seen in Fig 1A, there is no difference in appearance between the two capsule types (+/-DNA tagging).

All data presented here were obtained by March 2017, more than 2 years after completion of the 2014 tagging run, on tablets which has been stored continuously at lab ambient temperature.

In the present pilot study, the DNA tag was introduced into the pharmaceutical grade ink at a ratio of approximately 10^−9^ by mass relative to that of the ink itself. Given that the ink itself comprises only about 10^−7^ of the overall mass of the tablet, the resulting DNA/tablet mass ratio is thus approximately 10^−13^. The FDA and WHO both teach that DNA may be present in an oral dose at up to 100ug for residual DNA. Thus, given that these capsules weigh approximately 0.6 gram, the total DNA per capsule is approximately 10^-16^g, or about 10^−12^- fold below that FDA and WHO guidance and related FDA Guidance in, “Industry Incorporation of Physical Chemical Identifiers into Solid Oral Dosage Form Drug Products for Anti-Counterfeiting”, wherein DNA qualifies as a viable Physical and Chemical Identifier (PCID) under the 2011 FDA guidance. [12, 13, 14, 15, 16, 17, 18]

The DNA used to tag the subject acetaminophen capsules is below 200bp in length. We have shown here that such short DNA fragments can be sampled and authenticated via swabbing of the ink with an ethanol-moistened swab, followed by direct analysis of the swab-DNA complex, without DNA isolation or purification of any kind. We have shown that PCR-CE can be used on such swab isolates as a control assay to ensure that the DNA is in fact below the 200bp risk mitigating threshold as defined by the FDA and WHO, and to ensure the purity and identity of the DNA for the field-deployable nucleic acid analysis.

Fig 2 demonstrates that the DNA tag is indeed readily detectable by PCR-CE in the DNA tagged capsules via swabbing the “L” symbol or both the “L” and “5” symbols on the capsule surface. As expected, DNA is not detected in the untagged tablet control. Importantly, since sampling and analysis was performed after 2 years of tablet storage at laboratory ambient temperature these data demonstrate that the ambient-temperature shelf life of the DNA tag, as assessed by PCR-CE is greater than 2 years.

In addition to highly standardized DNA testing such as PCR-CE, in order to monitor a supply chain as complex as that in pharmaceutics, it is necessary to provide methods to sample and detect the DNA molecular tag in the field. Here, we describe the analysis of the DNA tag via two different methods of field-deployable nucleic acid analysis: isothermal amplification and qPCR. [25]

Isothermal DNA amplification is now widely deployed as a method of nucleic acid analysis in pathogen testing [26, 27, 28, 29, 30]. Fig 4 summarizes such field deployable DNA sampling and detection of DNA in the acetaminophen pilot study: via the sequence specific isothermal DNA amplification chemistry from TwistDx (Alere Corporation). [23] The Twist chemistry was deployed in the context of a portable device which can process up to 8 samples in parallel [24]. The readout from this device can be displayed as a real-time amplification curve or as a simple “plus”vs “minus” on the device. Both types of data are exportable to a laptop or PC and then to the internet to support data archiving and additional data analysis [23, 24]. In addition to portability, the instrument also has an open tangential optical path which makes it suitable for direct analysis of swabs and other solid materials.

Using the combination of Twist chemistry and the Axxin device, it is seen (Fig 4) that the same process of direct capsule swabbing, then analysis of the swab-DNA complex can cleanly detect the DNA tag in 20 minutes, under conditions where DNA in the matched, untagged control capsule is undetectable. Since sampling and analysis for isothermal amplification was also performed after 2 years of tablet storage at laboratory ambient temperature, these data demonstrate that the ambient temperature shelf life of the DNA tag, as assessed by sequence selective Twist isothermal amplification, is also greater than 2 years.

qPCR is also now widely deployed as a method of nucleic acid analysis for pathogen testing. [31, 32] Fig 3 displays a second approach to field-deployed DNA analysis, using instead the industry-standard sequence selective TaqMan qPCR assay as deployed on a small, portable qPCR device, which can process 16 samples in parallel [22]. The readout from the Mygo device is displayed as a real-time amplification curve on a laptop or PC, with ananalysis time of about 60 min. [22]

Using the combination of the TaqMan chemistry and the Mygo device, it is seen (Fig 3) that the same process of direct swabbing, then analysis of the unprocessed swab-DNA complex can cleanly detect the DNA tag by qPCR in these capsules in 60 minutes, under conditions where DNA in the untagged control is undetectable. Since sampling and analysis for TaqMan qPCR amplification was also performed after 2 years of tablet storage at laboratory ambient temperature, these data demonstrate that the ambient temperature shelf life of the DNA tag, as assessed by the qPCR is also greater than 2 years.

The data presented here show that a PCR generated DNA fragment may be used as a PCID (Physical Chemical Identifier) when added to an ordinary pharmaceutical capsule formulation as part of the food grade ink used as part of the capsule coating. The data show that, after 2 years of continuous storage at lab ambient temperature, the DNA tag, although introduced at only parts per billion (ppb) into the ink, can be collected by simple surface swabbing, then without subsequent DNA processing, the intact swab-DNA complex, can be analyzed by well-known methods of regulated lab based DNA forensics (PCR-CE) and also by the portable methods being developed for food safety, environmental screening and point of care diagnostics (isothermal amplification and qPCR). At the time writing this manuscript, the full commercialization of this technology is limited by the lead time for regulatory approval within the pharmaceutical and biotechnology industries. Activities are underway to seek industry approval [33]. Another consideration is that for eventual use in a regulated environment, neither the qPCR nor isothermal devices used in this study are 21 CFR Part 11 compliant, thus leaving a gap for Quality Control applications, while they can still be utilized in other industry applications [34].

In the aggregate, the data from this pilot suggest that DNA tagging can now become a routine component of pharmaceutical supply chain analysis: the goal being to augment better known print-based methods (like serialized bar coding) with the addition of DNA as part of the ink, or as an excipient more generally, to secure the authenticity of an individual drug formulation or dose from the manufacturer to the distributor to the pharmacy [35, 36].
